# ABMA: An Attention-Based Morphology-Aware Framework for Automated 12-Lead ECG Arrhythmia Classification

**DOI:** 10.3390/diagnostics16142274

**Published:** 2026-07-21

**Authors:** Manjur Kolhar, Raisa Nazir Ahmed Kazi

**Affiliations:** 1 Department of Health Information Management and Technology, College of Applied Medical Sciences, King Faisal University, Al-Ahsa 31982, Saudi Arabia; 2 Department of Respiratory Therapy, College of Applied Medical Sciences, King Faisal University, Al-Ahsa 31982, Saudi Arabia; rnahmed@kfu.edu.sa

**Keywords:** deep learning, electrocardiogram, morphology, attention layer, multi-class

## Abstract

**Background:** Cardiovascular diseases (CVDs) are among the leading causes of death globally. In order to treat CVDs successfully in the early stages, it is crucial to diagnose them in time. The ECG is one of the most common and non-invasive methods to detect heart rhythms and to diagnose arrhythmias. However, the analysis of ECG recordings manually requires a lot of time and experience because the morphology of ECG signals and the characteristics of their waveforms are very complex and show large overlaps between different types of arrhythmias. So far, various approaches for automated analysis of ECG signals have been developed, mostly based on deep learning (DL). In general, these methods are able to analyze ECG signals automatically and to detect different types of arrhythmias. Most approaches, however, are based on a purely data-driven feature learning and do not pay attention to the morphology-sensitive temporal structure of ECG signals, which is important for a discriminative diagnosis of arrhythmias. **Methods:** In this paper, we propose an Attention-Based Morphology-Aware (ABMA) framework to leverage multilead ECG signals in conjunction with automatically computed physiological features using a hybrid deep learning architecture. ABMA leverages multi-scale convolutional neural networks to learn local morphology features, and bidirectional long short-term memory (BiLSTM) networks to model temporal rhythms in ECG signals. We designed an ABMA module that incorporates a morphology scoring network (MSN) in order to (1) estimate the morphology-aware importance of different ECG segments and (2) learn the temporal importance of ECG features. The learned attention weights enable learning to focus on key sections of ECG signals without predefined boundaries or manual annotation of fiducial points. To understand the contribution of each individual component of the framework, we performed an extensive ablation study, where we removed the handcrafted feature branch, the ABMA module, the MSN, and the multi-head attention mechanism, one at a time, and compared the results against a fixed set of experimental configurations. **Results:** To assess the performance of the proposed framework in three-class classification between sinus rhythm (SA), atrial fibrillation (AFIB), and ventricular tachycardia (VT), we employed a stratified 10-fold cross-validation protocol. Our approach achieved a mean accuracy of 95.18 ± 1.18%, followed by a corresponding weighted F1-score of 95.19 ± 1.18% and a macro F1-score of 94.66 ± 1.35%. Notably, the performance of the proposed complete ABMA framework considerably outperformed the baseline CNN–BiLSTM architecture. Furthermore, in the primary evaluation metrics (i.e., accuracy, F1-score), the complete framework showed statistically significant improvements against the baseline through paired two-sided *t*-tests (*p* < 0.001). The ablation study indicated that each architectural component contributed positively to the overall classification performance, with the complete ABMA framework outperforming all reduced variants. **Conclusions:** The framework was evaluated by stratified cross-validation on a publicly available dataset. Our framework outperformed the baseline CNN–BiLSTM model as well as the respective ablation models in terms of classification performance. The findings from the current study are based on a retrospective analysis and therefore future studies using an independent external dataset, from multiple centers, or as part of a prospective clinical study are necessary in order to establish the generalizability and clinical utility of the proposed framework. The ABMA framework is currently viewed as a very promising research framework for intelligent ECG analysis, but it is not yet a clinically validated diagnostic tool.

## 1. Introduction

Cardiovascular diseases (CVDs) are among the top causes of death and illness worldwide. An effective and timely diagnosis is required [1,2,3,4]. The 12-lead electrocardiogram (ECG) is one of the commonly used non-invasive methods to diagnose cardiac arrhythmias and to facilitate clinical diagnosis [5,6,7]. However, manual analysis of ECG is very time-consuming and requires a high level of expertise. Moreover, the manual analysis of abnormal patterns in ECG signals is challenging due to their dynamic changes in shape and due to their strong overlap with various clinical conditions.

Deep learning (DL) techniques in the field of artificial intelligence (AI), which have developed rapidly in recent years have shown great success in the automatic analysis of the ECG. Convolutional neural networks (CNNs) and recurrent neural networks (RNNs) extract the hierarchical features of the ECG signal directly from the raw data [8,9]. Although predominantly data-driven models are being trained for the detection of arrhythmias, the learning of clinically relevant ECG waveform characteristics (e.g., P-waves, QRS-complexes, T-waves) is not encouraged [10,11,12]. On the other hand, the lack of interpretability of the models is a serious problem, especially in situations where the waveforms of the ECG signals are very similar to each other, such as differentiating sinus arrhythmia from atrial fibrillation. DL models have seen a recent surge in the use of an attention mechanism to decide which parts of the input signal are important and which are not. Most current attention mechanisms are data-driven, and attention to temporal information is directly learned from data for ECG classification. They are mostly designed to learn temporal importance in general, while learning representations of ECG signals, and do not explicitly investigate the design of morphology-sensitive feature weighting for learned ECG signal representations. Thus, the importance of different waveform regions is mainly determined through the optimization of classification instead of morphology-sensitive feature weighting. To overcome these limitations, this work explores the design of attention mechanisms that can morphologically adapt to and highlight important temporal diagnostic features of ECG signals. The current DL-based techniques for ECG classification have several shortcomings such as the lack of domain knowledge incorporated into the network and the lack of the important discriminatory information within ECG waveforms. The proposed Morphology-Aware Attention (ABMA) framework is integrated into the CNN–BiLSTM architecture using a dedicated morphology scoring network to learn the morphology-sensitive importance weights, and to enhance the temporal feature aggregation using the learned weights. In the introduced framework, deep neural networks are employed to utilize the raw 12-lead ECG signals as well as the manually crafted clinical characteristics extracted by techniques such as temporal features, morphology features and frequency features to classify the nature of an ECG signal, while the important morphology-guided weighting method is introduced in order to enhance the classification as well as to demonstrate the interpretability of the network. The proposed framework is evaluated and validated on a large-scale 12-lead ECG dataset and several external datasets. The experiments investigate a clinically meaningful multi-class classification problem of identifying sinus arrhythmia (SA), atrial fibrillation (AFIB) and ventricular tachycardia (VT) based on ECG recordings [12,13,14,15,16]. For this study, three types of arrhythmias were chosen: SA, AFIB and VT. All of these arrhythmias are generated by different electrophysiological mechanisms and show large differences in the temporal as well as the morphological development of their ECG signals. The SA rhythm is used as a physiological reference rhythm. The ECG of a patient with AFIB is highly variable not only in time but also in morphology, resulting in strongly irregular and variable RR intervals. Patients with a VT show an abnormal ventricular activation and an altered ventricular conduction. All three types of arrhythmias together serve as a test case for a morphology-sensitive ECG classification system, and, in addition, they are very common and dangerous in clinical applications. For this study, we used the following SNOMED CT codes to map the given diagnoses to: 427393009 for SA, 164889003 for AFIB, and 10370003 for VT. The given ECG signals that did not belong to any of the three target categories were removed from the datasets. We also removed all of the ECG signal files that were missing some critical diagnostic information, had incomplete metadata, were corrupted, and/or contained some form of annotation ambiguity. In order to evaluate the given ECG classification system in a manner that was both to be both robust and fair, we employed the given ECG signals in a stratified 10-fold cross-validation fashion. That is, all of the given ECG signal files for each of the given three categories of heart rhythm were divided into 10 nearly evenly sized groups, or folds. The key aspect of the given implementation of 10-fold cross-validation, however, was that we made every effort to keep the ratio of ECG signal files for each of the given heart rhythm categories in every fold of the ECG signal files as close as possible to the ratio of ECG signal files for each of the given heart rhythm categories in the overall dataset. This ensured that any given implemented ECG classification system would be tested on each of the given heart rhythm categories in turn, with the percentage of test ECG signal files of each heart rhythm category used in each fold equal to that category’s percentage in the overall dataset. In short, the 10-fold cross-validation used in this study provided a realistic and reproducible test of an ECG classification system that can take advantage of the two fundamental properties of ECG signals: their temporal properties and morphology-sensitive features.

To assess the robustness of ABMA, we have conducted a comprehensive set of experiments, including an in-depth analysis of an ablation study as well as cross-dataset studies. ABMA was found to effectively provide morphology-aware feature weights, which can provide complementary discriminative information for accurate ECG classification. Importantly, ABMA features favorable trade-offs between classification accuracy, transparency, and computational efficiency.

The main contributions of this study were summarized as follows:

1. In this paper, we present a novel Attention-Based Morphology-Aware (ABMA) framework for automated ECG arrhythmia classification. ABMA is different from existing self-attention mechanisms that compute similarity between query and key vectors to generate weights. In our framework, we incorporate a vectorized morphology scoring network that scores the morphology of all segments of the input ECG signal in a vector space, and then uses the network output to directly generate importance (attention) weights for all segments of the ECG signal in the latent space, where these morphology importance weights of all segments can be used to adaptively maximize the importance of informative segments and to minimize importance of less informative segments in aggregating features of all segments of the ECG signal.

2. We proposed a hybrid dual-input deep architecture that incorporates raw ECG signals with automatically extracted handcrafted physiological signal descriptors, in terms of rhythm, morphology and spectral features. The architecture, which is the first to seamlessly combine feature learning with physiological information, is capable of enhancing the classification performance of arrhythmic beats without having to manually extract ECG features.

3. A morphology-aware multi-head attention mechanism introduced along with temporal feature aggregation. The proposed module also employs parallel attention heads which learns complementary morphology-sensitive temporal representations through a dedicated morphology scoring network. This design enables more discriminative representations of ECG rhythm and waveform characteristics for robust classification.

4. We performed a comprehensive experimental evaluation using stratified 10-fold cross-validation, a statistical significance test and an extensive ablation study. In the ablation study, we quantified the contribution of the handcrafted feature branch, the Morphology-Aware Attention module, the morphology scoring network, as well as the multiple heads of attention. All components were found to be individually contributing to the performance of the classifier.

5. We also evaluated the proposed framework on several external public datasets, namely PTB-XL, Georgia and CPSC-2018. The performance on the proposed internal classification task was satisfactory. However, the external experiments clearly show the variability between the given datasets and therefore the challenge of cross-dataset generalization. Future work has to address the issue of domain adaptation, feature recalibration, transfer learning and multi-source learning in order to apply the approach in a clinical setting.

The remaining sections are Methods, Materials and Experimental Setup; Dataset; Feature Engineering and Model Pipeline; and Experimental Setup and Statistical Analysis. We present the demographic and clinical characteristics of our dataset, and then thoroughly investigate the performance of the proposed solution under various settings. We also discuss the results of the feature selection process, comparison studies, clinical implications and consequences of our findings. Finally, an ablation study on the contribution of the Morphology-Aware Attention (ABMA) component to the accuracy of the classifier is provided.

## 2. Methods

### 2.1. Study Design

**Figure 1:**
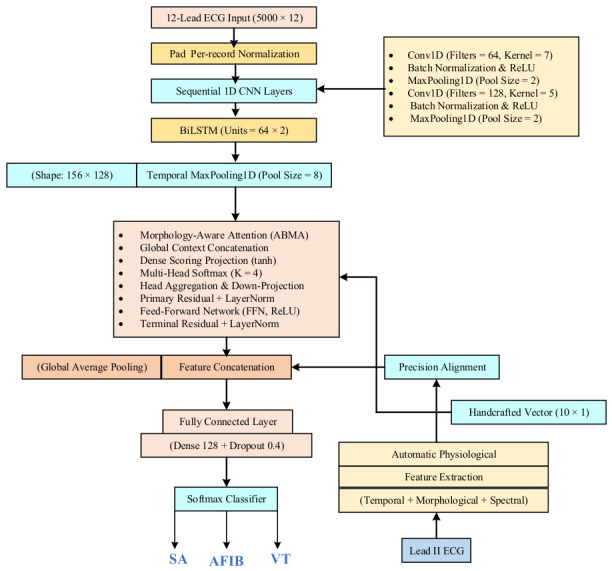
Proposed model.

This study uses a publicly available 12-lead ECG database for the development and evaluation of a deep learning framework for arrhythmia classification. The framework combines a set of handcrafted clinical features for ECG with features learned from the data. To this end, an Attention-Based Morphology-Aware (ABMA) module is proposed. The ABMA module includes a dedicated morphology scoring network that learns morphology-sensitive importance weights, denoted as attention weights, from intermediate feature representations of ECG features. Unlike standard attention, which learns temporal importance solely through optimization, the weights learned by the ABMA module can be used to adaptively re-weight the feature representations of different time instants in accordance with their learned diagnostic relevance, thereby improving temporal feature aggregation and model interpretability. The overall architecture of the proposed framework is depicted in Figure 1.

The ECG Big Data Analytics Challenge dataset was used for model development and evaluation. In this study, a clinically meaningful three-class classification problem was considered, consisting of sinus arrhythmia (SA), atrial fibrillation (AFIB), and ventricular tachycardia (VT). These arrhythmias were selected because they represent distinct electrophysiological mechanisms and exhibit substantially different temporal and morphological ECG characteristics. To enable a focused and controlled evaluation of the proposed ABMA mechanism, all experiments were conducted using this three-class classification setting.

### 2.2. Model Architecture

To integrate the automatically extracted physiological descriptors from the ECG signals with the deep features that have been learned from the ECG signals, we have developed a dual-input deep learning framework. The new framework consists of two new branches, namely the deep signal processing (DSP) branch and the auxiliary feature (AF) branch. The signal branch of the framework is designed to take the entire 12-lead ECG signal as input and to learn deep features from the multi-channel ECG signal. On the other hand, the auxiliary feature branch of the framework is designed to automatically extract the handcrafted, physiological descriptors from the corresponding ECG Lead II signal. Prior to the feature extraction by the two branches of the framework, the entire ECG signal of interest is padded with zeros to a fixed length of 5000 samples. The padded length of 5000 samples is then used as the input to the two branches of the framework [17]. After feature extraction by the two branches of the framework, the handcrafted physiological descriptors are normalized (i.e., standardized) to zero mean and unit variance. The normalized handcrafted descriptors are then used as the input to the subsequent layers of the network. The overall framework of the newly proposed ABMA model is depicted in Figure 1. The auxiliary feature branch of the network is utilizing 10 fully automated, delineation-free physiological descriptors extracted from the aligned Lead II ECG signal [18,19]. These descriptors are grouped into three different categories. The first category consists of four rhythm descriptors: the mean RR interval ($RR_{\mathrm{mean}}$), the standard deviation of RR intervals RR interval $\left(RR_{\mathrm{std}}\right)$ the root mean square of RR intervals RR interval ($RR_{\mathrm{rms}}$), and the RR irregularity ratio RR interval ($RR_{\mathrm{irreg}}$). These features can be used to assess heart rate variability as well as rhythm irregularity and are thus particularly well suited to distinguish between atrial fibrillation and normal sinus rhythm. The second category consists of two QRS morphology proxy features, i.e., the mean and the standard deviation of the full width at half maximum (FWHM) of the individual R-peaks detected in the ECG signals. These features provide an approximate representation of the ventricular depolarization morphology without the need to individually identify the corresponding QRS onsets and offsets. In this work, we use these simple features as efficient QRS morphology proxies, fully compatible with the provided manual feature extraction framework.

The third group consists of 4 global spectral descriptors, i.e., the mean, standard deviation, skewness and kurtosis of the FFT magnitude spectrum of the ECG signal. The proposed feature extraction in this paper does not need ECG delineation or fiducial-point annotation. Therefore, there is no extraction of PR-interval, ST-segment, P-wave or T-wave features. As a result, the proposed processing pipeline offers fully automated, operator-independent and reproducible ECG signal processing for the extraction of ten different physiological descriptors. The deep signal branch starts with a number of layers of convolutional feature extractors, followed by a CNN–BiLSTM backbone. The convolutional layers are able to learn local properties of the ECG signal, while the bidirectional long short-term memory (BiLSTM) layer is able to learn long-term dependencies in the signal and form an understanding of the rhythm and dynamics of the signal over time. In order to further enhance the temporal feature learning of the signal, an Attention-Based Morphology-Aware (ABMA) module is introduced and placed after the CNN–BiLSTM backbone. In contrast to typical implementations of temporal attention and multi-head self-attention that learn attention weights based on the similarity between query, key and value, the novel ABMA module is equipped with a dedicated morphology scoring network that directly learns temporal saliency on a position-wise basis from the underlying CNN–BiLSTM feature representations. In addition to the feature representations, the handcrafted descriptor vector, which is standardized to have the same range, is broadcast along the temporal axis and concatenated with the feature sequence. The morphology scoring network learns to estimate the temporal importance jointly from the deep features and the explicit features of the rhythm, the QRS-width proxy and the spectral features. Temporal Adaptive Weighting Networks are used to adaptively reweight the temporal feature sequence to emphasize the most diagnostically informative parts of the ECG signal over time. To learn different temporal saliency, multiple attention heads are used, and the outputs of the attention heads are projected back to the feature dimension of the input feature sequence by a linear layer and then all the attention head outputs are concatenated, projected back down to the original feature dimension space and added to the input feature sequence with a residual connection.

### 2.3. Feature Extraction Strategy

To further improve the discriminative ability of the proposed framework, a dual-input framework that combines domain-informed handcrafted features with data-driven CNN–BiLSTM representations (Table 1) was employed. For each ECG recording, ten fully automated descriptors were extracted from the raw Lead II signal after per-record z-score normalization of the raw recordings. These explicit physiological descriptors are highly complementary to the automatically learned deep sequence features and allow the model to simultaneously exploit clinically relevant rhythm information as well as automatically detected temporal patterns. Consequently, the ABMA module is conditioned on the deep feature representations as well as on the descriptors for rhythm-, morphology- and frequency-related information of the signal. Thus, the temporal features are improved by aggregating more information while keeping the analysis fully automatic; refer to the Algorithm 1 for the feature extraction pipeline [20,21,22,23,24,25,26,27,28,29,30].
**Algorithm 1.** Automated non-Delineation Feature Extraction PipelineInput: Raw ECG data$X\in R^{T\times 12}$Output: $F\in R^{10}$
Step 1: lead selectionLead II is extracted$x=X_{:,2}\in R^{T}$Whereas $X_{:,2}$, represents second column for any given row for *T* samples. Therefore $x=R^{T}$. The signals are resized $s=\mathrm{Resize}\left(x,L\right),s\in R^{L},$ where $\mathrm{Resize}\left(\cdot \right)$ denotes truncation for signals longer than *L* samples and zero-padding for shorter signals.Step 2: Automatic R-Peak Detection$d_{min}=max\left(1,\;\left\lfloor \frac{f_{s}}{2}\right\rfloor \right)$, located in $R=\left\{r_{1},r_{2},r_{3},\ldots r_{p},\right\},\;r_{p}\in \left\{1,\ldots L\right\},$Step 3: RR Rhythm DescriptorsIf P≥2, RR interval vector$q={\left[q_{1},q_{2},q_{3},\;\ldots q_{N}\right]}^{T}\in R^{N}$For $q_{i}\frac{r_{i+1}-r_{i}}{f_{s}}$ $i=\left\{1,\ldots ,N\right\}$
All RR Descriptors$F_{1}=\mu_{q}=\frac{1}{N}\sum_{i=1}^{N}q_{i},$ $F_{2}=\sigma_{q}=\sqrt{\frac{1}{N}\sum_{i=1}^{N}(q_{i}-\mu_{q}{)}^{2}},$ $F_{3}=RMS\left(q\right)=\sqrt{\frac{1}{N}\sum_{i=1}^{N}q_{i}^{2}},$ $F_{4}=\frac{1}{max(1,N-1)}\sum_{i=1}^{N-1}I\left(|q_{i+1}-q_{i}|>0.05\right),$If P<2,$F_{1}=F_{2}=F_{3}=F_{4}=0.$Step 4: QRS-Width Proxy DescriptorsIf P≥1, the peak-width vector is$w=[w_{1},w_{2},\ldots ,w_{K}{]}^{T}\in R^{K},$where each width is estimated at half-maximum peak prominence. The extracted descriptors are$F_{5}=\mu_{w}=\frac{1}{K}\sum_{k=1}^{K}w_{k},$ $F_{6}=\sigma_{w}=\sqrt{\frac{1}{K}\sum_{k=1}^{K}(w_{k}-\mu_{w}{)}^{2}}.$If no valid widths are available,$F_{5}=F_{6}=0.$These are QRS-width proxy descriptors. As such, they do not rely on explicit delineation of the onset and offset of the QRS complexes.Step 5: Global Spectral DescriptorsThe FFT magnitude spectrum of the aligned ECG signal is$m=|F(s)|=[m_{1},m_{2},\ldots ,m_{L}{]}^{T}\in R^{L}.$The extracted spectral descriptors are$F_{7}=\mu_{m}=\frac{1}{L}\sum_{j=1}^{L}m_{j},$ $F_{8}=\sigma_{m}=\sqrt{\frac{1}{L}\sum_{j=1}^{L}(m_{j}-\mu_{m}{)}^{2}},$ $F_{9}=\mathrm{skew}\left(m\right),$ $F_{10}=\mathrm{kurtosis}\left(m\right).$If$\sigma_{m}=0,$then$F_{9}=F_{10}=0.$Step 6: Feature Vector AssemblyThe final handcrafted descriptor vector is$F={\left[\begin{array}{cccccccccc} F_{1} & F_{2} & F_{3} & F_{4} & F_{5} & F_{6} & F_{7} & F_{8} & F_{9} & F_{10} \end{array}\right]}^{T}\in R^{10}.$

### 2.4. Morphology-Aware Attention (ABMA)

The Attention-Based Morphology Alignment (ABMA) module is developed to be placed after the deep spatiotemporal backbone in order to enhance the classification performance for SA, AFib, and VT. Unlike standard multi-head self-attention, where sequence dependencies are computed by similarity internal search (i.e., (Query×Key) product), the external-context-conditioned additive scoring architecture is developed to isolate critical morphological variations in temporal domain and to simultaneously map global contextual markers. The important weights for different segments are assigned by an explicitly trained Feed-Forward Network with non-linear mapping, where informed segments that are critical for diagnosis are emphasized, whereas noisy or non-informative regions are discarded.

The input to the ABMA block is the spatiotemporal latent sequence produced by the down sampled multi-scale CNN and BiLSTM layer stacks. We refer to this sequence as the following matrix:

$$X=[x_{1},x_{2},\ldots ,x_{T}{]}^{T},X\in R^{T\times d}$$

Whereas T = 156 denotes the down sampled number of temporal steps, and d = 128 is the hidden dimension of the features in the latent embeddings.

In order to keep the structural complexity constant and to only evaluate the diagnostic weight of a certain feature, a vectorized scoring network is used to process all time steps and all attention heads of a network simultaneously. The latent sequence of a network is enriched by a global handcrafted morphological vector of size 10 $(F\;\in \;R^{10}$) that contains information about the global rhythm of a sequence. This global vector is added to the context steps of the latent sequence to form a context matrix $Z_{\left\{t\right\}}=\;\left[x_{\left\{t\right\}}∥\;F\right]$. The scoring engine of the vectorized scoring network then uses two densely connected layers with activation function tanh to score all context steps of the enriched context matrix $Z_{\left\{t\right\}}$ of a network:

$$S=\tanh\left(ZW_{1}+b_{1}\right)W_{2}+b_{2}$$

Let $W_{1}$ denote the weight matrix for the intermediate hidden projection and the corresponding bias vector. Meanwhile, $W_{2}$ projects the feature space directly to the attention heads and $b_{2}$ is the head bias. Importantly, this vectorized projection is jointly generated for all temporal positions, yielding raw, unscaled importance scores S for each of the K=4 attention heads.

The raw diagnostic score of hidden sequence $S_{t}$ is transformed into a temporal probability distribution in the timeline by applying Softmax activation function exclusively over the temporal dimension (axis = 1) to get a scaled attention weight $A_{h,t}$ for an individual attention head $h\in \left\{1,\ldots ,K\right\}$ at time-step t.

$$A_{h,t}=\frac{\exp\left(S_{t,h}\right)}{\sum_{j=1}^{T}\exp\left(S_{j,h}\right)},\;\mathrm{subject}\;\mathrm{to}\;\sum_{t=1}^{T}A_{h,t}=1$$

The weights obtained for a single attention head at a specific time step of the hidden sequence constitute a localized diagnostic filter that highlights highly characteristic signs of rhythm instability or wave distortion by taking large values in the corresponding time steps.

To preserve independent tracking criteria for the multiple tasks, the learned attention weights are broadcasted along the feature rows of the native latent sequence X to yield a set of localized representations, each corresponding to a single head h, and given by:

$$H_{h}=X⊙\mathrm{Tile}\left(A_{h}\right)\in R^{T\times d}$$

Note that ⊙ denotes element-wise scaling. The morphological structures synthesized to evaluate different heads of a model are synthesized in parallel and, hence, concatenated along the feature dimension, and then are projected back onto the original feature space. To counteract vanishing gradients and to foster smooth representations of samples in the model, the tensors resulting from the attention fusion are processed in a robust residual processing stream. For this purpose, the features already processed by the BiLSTM sequence are normalized on the basis of the corresponding projected features in the first track of the Layer Normalization, into which a corresponding dropout operation (p=0.1) is integrated.

$$X_{\mathrm{attn}}=\mathrm{LayerNorm}\left(X+\mathrm{Dropout}\left(X_{\mathrm{proj}}\right)\right)$$

After normalization, the intermediate sequences are then passed through a position-wise Feed-Forward Network (FFN) containing a Rectified Linear Unit (ReLU) activation to introduce non-linear depth.

$$\mathrm{FFN}\left(X_{\mathrm{attn}}\right)=\mathrm{ReLU}\left(X_{\mathrm{attn}}W_{f1}+b_{f1}\right)W_{f2}+b_{f2}$$

The internal dense layers are of capacity of 128 hidden channels. A secondary residual connection adds the FFN output to the final output, resulting in the unified morphology-enhanced output Y:

$$Y=\mathrm{LayerNorm}\left(X_{\mathrm{attn}}+\mathrm{Dropout}\left(\mathrm{FFN}\left(X_{\mathrm{attn}}\right)\right)\right),\;Y\in R^{156\times 128}$$

### 2.5. Dataset

The ECG data used in this paper is a subset of the publicly available Chapman–Shaoxing–Ningbo ECG database developed by Chapman University, Shaoxing People’s Hospital, and Ningbo First Hospital and released through PhysioNet [31]. All the experiments were conducted on a local copy of the database. The database consists of a total of 45,152 standard 12-lead ECG recordings, each sampled at 500 Hz. The corresponding diagnostic statements were annotated by cardiologists from three hospitals and expressed in SNOMED CT. The abnormalities in the ECG data cover a wide spectrum of heart diseases including arrhythmia, conduction disorder, abnormal sinus rhythm, myocardial infarction, hypertrophy and ST/T-wave abnormalities.

This study is designed as a three-class classification problem for recognizing rhythm of SA, AFIB, and VT. These heart rhythm disorders were chosen because they are clinically significant, have different temporal characteristics and ECG morphology, and are challenging for classification using morphology-aware attention. By limiting the study to these three types of heart rhythms, we can compare the performance of our method with other classification methods without introducing additional complexity caused by the large number of different cardiac conditions and rhythms that are present in the full database.

We used a predefined mapping to extract the corresponding diagnostic labels from the available SNOMED CT annotations. For the purpose of this study, we have excluded all records which correspond to a set of diagnostic classes not included in the three rhythm classes of interest. As the available Chapman–Shao database consists of a large number of ECG recordings each having multiple diagnostic statements (annotated by a physician) corresponding to a record, we have created a single-label classification problem (a record corresponds to a single class) by following a predefined diagnostic priority order for the records corresponding to multiple rhythm classes (VT > AFIB > SA). Thus, all eligible recordings have been included in this study. The summary of the study data is provided in Table 2 where it can be seen that a total of 3740 ECG records have been included in the study with 2671 of them corresponding to SA (71.4%), 804 to AFIB (21.5%), and 265 to VT (7.1%).

Given the relatively small number of recordings available for the VT task, an imbalanced dataset would have posed a significant challenge in terms of avoiding any form of bias in the modeling process and subsequent performance estimation. To address this, a number of important steps were taken within the validation framework. The 20 recordings were split using stratified 10-fold cross-validation to ensure that performance was tested and reported on using a number of folds that included an equivalent number of minority class recordings (those corresponding to SCD) through all subsequent stages of testing and training. Subsequently, within each of the 9 folds used for model training and testing (one fold was set aside for independent testing as before), a stratified validation subset of 17.65% of the recordings was created and used for hyperparameter search, model selection and for driving the early stopping criterion. The resulting percentages for the 3 sets of data (i.e., training, validation, test sets) were approximately 74%, 16% and 10%, respectively. Importantly, the independent test set (corresponding to one of the folds created by 10-fold cross-validation) had not been used for or exposed to modeling in any form during the entire development process.

To prevent information leakage, we partitioned data at the patient level and ensured that all preprocessing, feature extraction and feature standardization were done within each cross-validation iteration separately using only statistics and data from the corresponding training partition. The learned normalization parameters were applied to the validation and test partitions subsequently. The final dataset was still imbalanced, especially for ventricular tachycardia. To deal with this imbalance, all measures were taken during the training of the model. An online data augmentation pipeline, for example, amplifies records slightly, adds Gaussian noise and shifts the start of records, all in a mild way, in order to increase the amount of training data and to increase diversity, while keeping the underlying physiology of the records intact. Additionally, a class-balanced multi-class focal loss is used. This loss function reduces the impact of easy majority-class records and increases the impact of all minority-class records. As with the data augmentation, no data augmentation and no focal loss weighting were done for the validation dataset and the test dataset, i.e., all measures were performed on data that the model had not seen before and that was not changed in any way. Thus, all measures presented here are valid for records that the model has not seen before. Instead of reporting overall accuracy on the imbalanced dataset, we provide a more balanced assessment of model performance. In addition to accuracy, we report precision, recall, F1-score, macro-averaged F1-score, weighted F1-score, ROC-AUC and Precision-Recall AUC (PR-AUC) for all classes. The last two metrics in particular are of high importance when dealing with imbalanced datasets, especially in the case of ventricular tachycardia. For all experiments, a fixed random seed of 42 was used to make the partitioning of the data deterministic and reproducible. All models have been evaluated with the same data preprocessing, cross-validation, hyperparameter optimization and evaluation metrics. Thus, the CNN–BiLSTM baseline, as well as the proposed ABMA framework and all the ablation models are compared on an equal footing.

### 2.6. Experimental Setup

**Figure 2:**
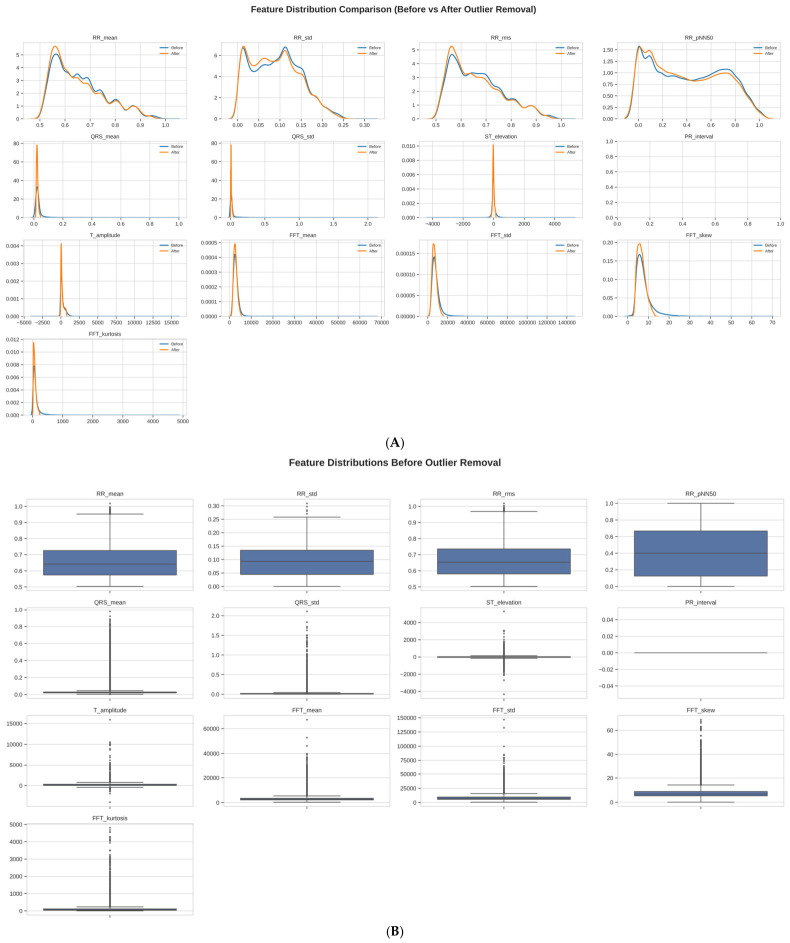

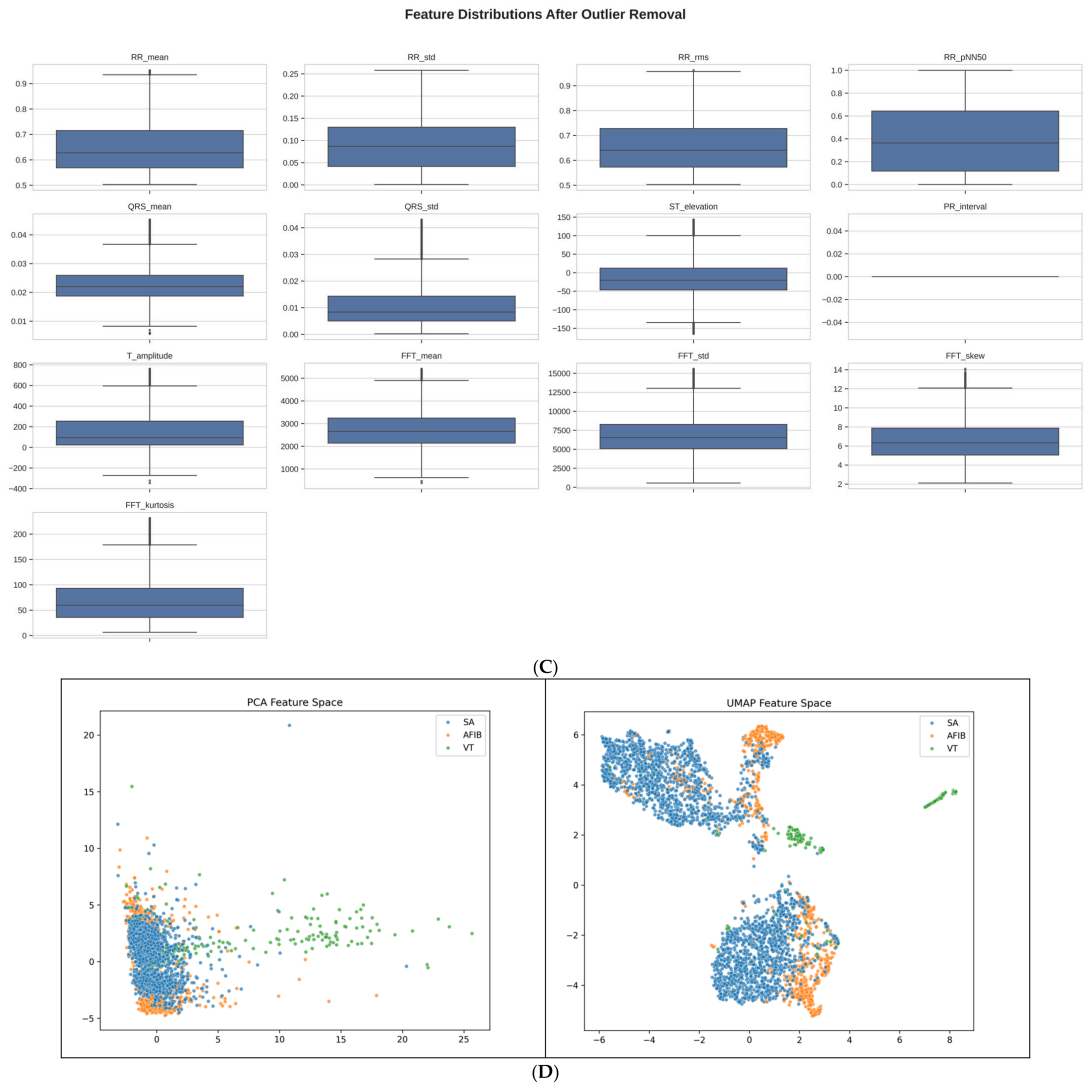
(**A**) Kernel density plots of feature distributions before and after outlier removal. (**B**) Feature distributions before outlier removal. (**C**) Outlier removal on feature distributions. (**D**) PCA and UMAP projections of features learned by the proposed ABMA model, showing improved separation of SA, AFIB, and VT classes in the latent feature space.

Figure 2 presents the three diagnostic classes analyzed in the current study. All experiments were conducted with the same protocol across all folds of the 10 stratified cross-validations. In each of the 10 cross-validation iterations, one fold was used for testing, while the other 9 folds were used for building a model. A validation subset was also created from the training data partition for model optimization purposes, such as for early stopping. The preprocessing of the data, as well as the standardization of the features, were performed using statistics calculated from the corresponding training data partition only. Thus, no information from the validation or testing data was introduced into the model.

To combat imbalanced data, in particular the few VT samples, we employed online data augmentation and optimization. First, we perform a random on-the-fly data augmentation of the training data. Every ECG is then randomly scaled in amplitude by 10% up and down, Gaussian noise of a fixed but small standard deviation of 0.02 is added, and the ECG is then shifted in time by a random number of samples, up to 25 samples in either direction. This way, we significantly increase the number of samples in the training data, but the ECG’s spatiotemporal characteristics remain preserved. For the optimization, we then abstain from structural oversampling as well as from boundary cleaning, which in both cases would result in the network being exposed to the same, possibly even noisy, ECG border samples over and over again. Instead, we tackle the class imbalance within the loss function, by using a multi-class variant of the focal loss, where every class receives a weight inversely proportional to its frequency in the current fold, and a focusing parameter of γ = 2.0, which causes the loss function to focus even more on the hard samples of the minority classes and less on the easy samples of the majority classes. We do not apply any data augmentation or focal-loss weighting to the validation set and test set. All of the evaluation metrics that we report are computed on previously unseen, unprocessed ECGs, providing a fair assessment of the proposed model.

The model parameters are optimized using the Adam optimizer for mixed-precision training, starting with a learning rate of $3\times 10^{-4}$. A two-phase WarmupCosineDecay learning-rate schedule was used to stabilize the optimization in the initial training phase. The schedule first increased the learning rate from the initial value, over 5 warmup epochs, and then applied cosine annealing until 60 epochs of training had been reached. The schedule then terminated at the minimum learning rate of $1\times 10^{-6}$. To improve generalization we added dropout regularization to the attention and classification layers. A dropout rate of 0.1 is used within the morphology-aware attention module. A dropout rate of 0.2 is used within the Feed-Forward Network (FFN). A dropout rate of 0.4 is used before the classification layer to avoid overfitting and enable generalization. For early stopping, we monitored the validation loss with a patience of 10 epochs. The model was then restored to the weights before the patience was reached for each epoch where the model had the lowest validation loss. The performance of the final model was then measured and reported as the mean and standard deviation of the accuracy, weighted F1-score, macro F1-score, and the class-wise F1-scores. In addition, the performance was also reported as the receiver operating characteristic area under the curve (ROC-AUC) and the training time in seconds for each of the 10 cross-validation folds.

To quantify the contribution of the principal architectural components of the proposed framework, a comprehensive ablation study was performed. All experiments were conducted under identical setup for a stratified 10-fold cross-validation. Four different reduced architectures were evaluated against the full ABMA model. All other parts of the network, the training setup, and the hyperparameters were left unchanged in order to ensure a valid comparison. First, we ablate the handcrafted feature branch to train the model to directly classify from the deep representations learned from raw multilead ECG signals. This helps us to understand the contribution of auxiliary physiological descriptors as features on the classification performance. Second, we ablate the ABMA module to understand the importance of ablation of attention to locally emphasize the correct diagnostically relevant parts of ECG morphologies. Third, we ablate the morphology scoring network, keeping the rest of the attention mechanism intact to find out the contribution of morphology-aware importance estimation for feature refinement. Lastly, we ablate the four-head attention in the proposed architecture and replace it with a single-head attention to understand whether learning of multiple complementary morphology representations using multi-head attention is beneficial. All ablation experiments were set up to use the exact same data partitioning, preprocessing, optimization, learning-rate scheduling, and evaluation methodology. Thus, all differences in performance between the ablated versions and the complete system can be unambiguously attributed to the removed architectural component. Results of the complete ablation study are presented in Section 3.4 and allow for a quantitative assessment of the individual contributions of all single components to the overall performance of the proposed ABMA framework.

### 2.7. Statistical Analysis

To assess the quality of our novel ABMA framework, we employ a set of standard quality measures for classification, i.e., accuracy, precision, recall, and F1-score In cases where one of the categories is more frequent than the others, precision and recall are biased towards the more frequent category. To provide a more balanced view, we compute the F1-score, which is the harmonic mean of precision and recall. We provide a summary of the classification results by means of confusion matrices, which also illustrate the number of false positives and false negatives. Moreover, the receiver operating characteristic (ROC) curves with the corresponding area under the curve (AUC) values measure the ability of our models to distinguish between classes. We use paired *t*-tests to test for significant differences between the models on all folds.

The mean RR interval ($RR_{mean}$) was the most informative statistic to characterize the rhythm of the different arrhythmias. The variability of irregularly changing beat to beat intervals of AFIB recordings was in stark contrast to the highly rapid and regular ventricular activation of VT recordings. The spectral complexity of the recordings was characterized by the FFT kurtosis ($FFT_{kurtosis}$) which for VT recordings yielded sharp peaks reflecting the rapid and relatively regular ventricular activity (Figure 2A–C). Here, we look at the distributions of the ECG-derived features before and after data cleaning in a kernel density estimation plot (Figure 2A) and a boxplot (Figure 2C). The distributions of the cleaned data have lower variance and are smoother than the distributions of the features before data cleaning. They thus constitute a more stable and less dispersed training set. The distributions before data cleaning are skewed, have heavy tails and extreme values.

To assess class separability of the ECG features, we reduced the dimensionality of the feature space using two methods: Principal Component Analysis (PCA) and Uniform Manifold Approximation and Projection (UMAP). From the PCA feature space, it is clear that while there is considerable overlap between the SA and AFIB classes, the VT class forms distinct morphological patterns that are easily separable from the other two classes. On the other hand, the UMAP feature space shows improved class separability that improves across the three classes. The VT class clusters are well separated from the other two classes. However, the samples from the AFIB class form an intermediate region between the two clusters formed by the SA and VT classes. This intermediate region of the AFIB class implies the existence of non-linear relationships in the ECG feature space that can be modeled by the CNN–BiLSTM architecture in conjunction with the ABMA framework (Figure 2D).

## 3. Results and Discussion

### 3.1. Demographic and Clinical Findings

Table 2 shows the final study cohort consisting of a total of 3740 ECG recordings. These recordings were classified into three different types of rhythm disorders, i.e., SA, AFIB and VT. In detail, there were 2671 recordings of SA, 804 recordings of AFIB and 265 recordings of VT. Table 2 presents an overview of the study cohort. Note that SA accounted for the majority of the recordings (71.4%), followed by AFIB (21.5%) and VT (7.1%). The mean age of the patients was 42.17 ± 26.01 years. The patients with VT were the oldest (71.97 ± 10.66 y), followed by the patients with AFIB (62.66 ± 16.85 y), while the patients with SA were the youngest (33.05 ± 23.52 y). There was nearly a balance of males and females in the database. There were 1948 males (52.1%) and 1792 females (47.9%) in the database. While there was nearly a balance of males to females with SA, there were significantly more males with AFIB and VT than females. All recordings were sampled at 500 Hz, and all recordings were of a length of 5000 samples for the model. A large amount of variance is introduced by the age distribution and the characteristic rhythms for the three types of arrhythmia, i.e., SA, AFIB, and VT. These differences will be used to test the proposed approach ABMA in a three-class classification problem. Age and sex information were reported for cohort characterization only and were not used as input variables during model training.

### 3.2. Baseline Model Performance

**Figure 3:**
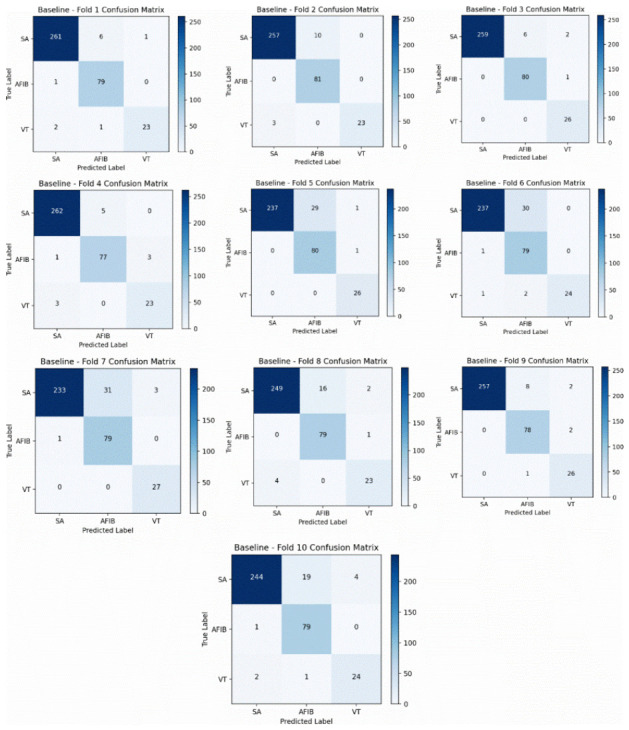
Confusion matrices of the baseline model across all 10 folds, illustrating class-wise classification performance for SA, AFIB, and VT.

**Figure 4:**
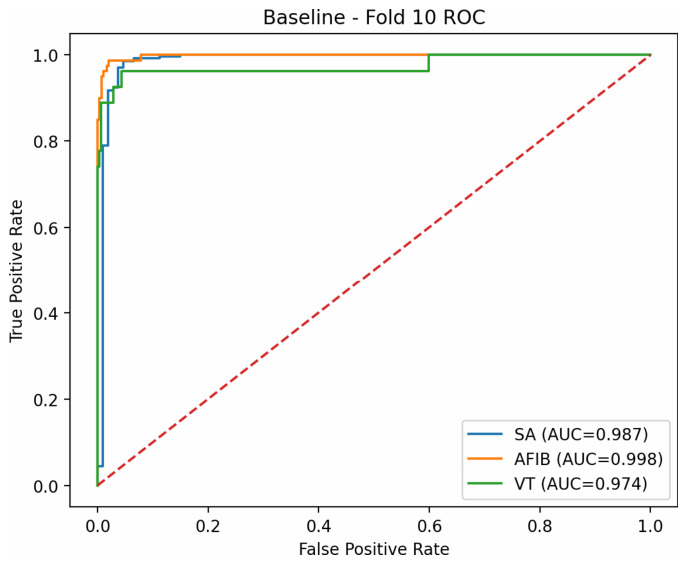
ROC curves of the baseline model for SA, AFIB, and VT classes with corresponding AUC values.

In this section, we first test the performance of the baseline CNN–BiLSTM model without any attention mechanism. For this purpose, we use stratified 10-fold cross-validation and present the corresponding training loss curves, confusion matrices and ROC curves in Figure 3 and Figure 4. The corresponding quantitative results are listed in Table 3.

The baseline architecture, CNN–BiLSTM, serves as a reference point for the subsequent studies. We compared the performance of all individual classes (SA, AFIB, VT) by reporting the performance by a set of standard metrics, i.e., accuracy, a weighted (F-1)-score and a macro (F-1)-score. Finally, we also reported on the performance in terms of computational efficiency by recording the training time for each fold as well as the test-partition inference time per fold in the test partition.

In Table 3, the performance of the baseline model is detailed. This model already achieved a very good performance with a mean accuracy of (94.44 ± 2.73%) and a weighted (F-1)-score of (94.60 ± 2.54%). Also, the corresponding macro (F-1)-score reached a high value of (92.77 ± 2.27%) because of the imbalanced distribution of the classes in the dataset. This was even harder to achieve for the model than the previously mentioned scores. The average training time per fold for this model was (3429.96 ± 1059.49) s while the average time for classification on the test set (after training) was (1.83 ± 0.20) s. Qualitative assessment of the ability of the baseline framework to distinguish between the three classes of cardiac rhythms demonstrated good performance in classifying SA rhythms with the majority of SA recordings being correctly classified. The performance for the classes of recordings corresponding to AFIB and VT were, however, more mixed, with greater levels of misclassification between the two classes. The corresponding ROC curves in Figure 4 show good discriminative performance for all classes with ROC–AUC values greater than 0.90 for all classes. Despite stable performance across folds, the performance on the AFIB class was worst with a mean (F-1)-score of (87.26% ± 7.22%) and the largest interfold variance. The difficulty to distinguish AFIB from SA and VT likely stems from the often heterogeneous rhythm and morphology within atrial fibrillation recordings. The baseline CNN–BiLSTM architecture processes all temporal feature representations equally without incorporating attention or morphology-aware feature weighting. As a result, all temporal regions contribute equally to the feature aggregation, and therefore, diagnostically informative regions have the same impact as less informative regions. Experiments to investigate the use of attention and morphology-aware feature weighting were thus conducted in the following (Refer Figure 3 and Figure 4).

### 3.3. Morphology-Aware Attention (ABMA) Model Performance

**Figure 5:**
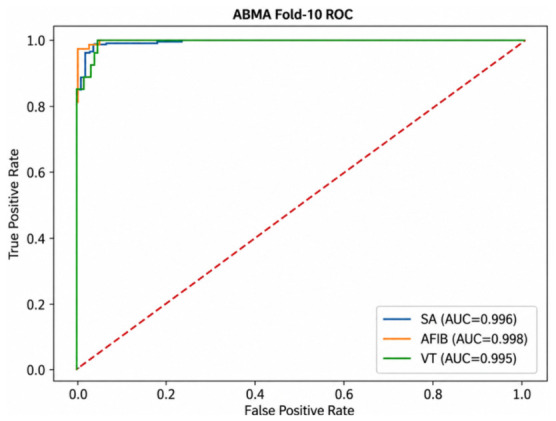
ROC curves of the ABMA model showing class-wise performance for SA, AFIB, and VT with corresponding AUC values.

**Figure 6:**
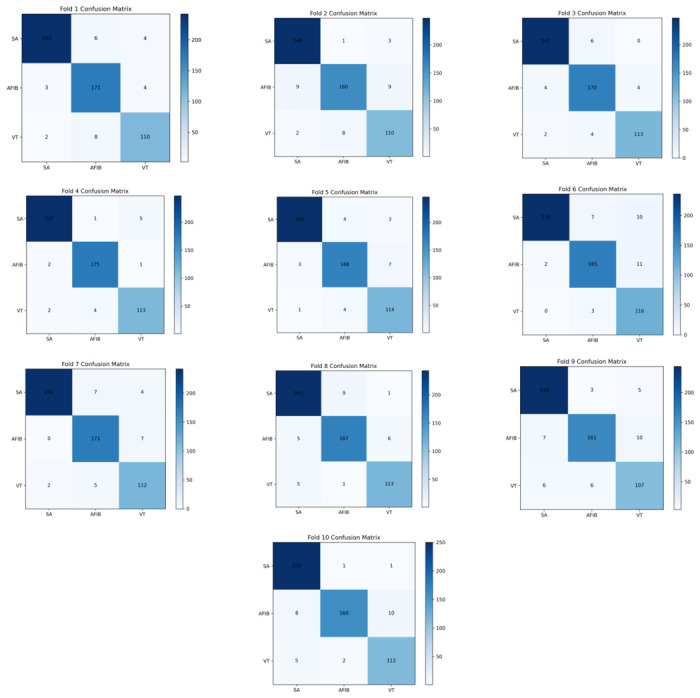
Confusion matrices across 10 folds for the ABMA model.

The results of 10-fold cross-validation experiments for the ABMA model are shown in Table 4. The corresponding ROC curves for all classes are shown in Figure 5, and the corresponding confusion matrices are shown in Figure 6. The ABMA model achieved a mean classification accuracy of 95.18 ± 1.18%, a weighted F1-score of 95.19 ± 1.18%, and a macro F1-score of 94.66 ± 1.35%. The class-specific mean F1-scores were 97.00 ± 0.76% for SA, 94.28 ± 1.54% for AFIB, and 92.70 ± 2.16% for VT. The highest classification accuracy was achieved in Fold 4 with accuracy of 97.27% and a macro F1-score of 96.91%, while the lowest accuracy was achieved in Fold 9 with accuracy of 93.26% and a macro F1-score of 92.40%. Moderate variation in accuracy was observed between the folds; however, the standard deviations are relatively small, indicating that the model generalizes well and has stable performance. In Figure 5, we show the ROC curves for the best fold, which corresponds to the highest performance of the ABMA framework on the SA, AFIB and VT classes. For this fold, the one-vs-rest ROC-AUC values are equal to 0.9940 for SA, 0.9955 for AFIB and 0.9963 for VT classes, meaning that the framework is able to classify the different cardiac rhythms very well. The good performance and the ability of the framework to classify between the three classes are also demonstrated by the high ROC curves, close to 1, obtained for all the folds, as shown in the figure. In comparison to the baseline CNN–BiLSTM model, the results for the ABMA framework were generally improved in terms of overall accuracy as well as the weighted F1-score and the macro F1-score. In comparison to the model without the Morphology-Aware Attention module, the improvements were, however, relatively small. In particular, the improvements for the AFIB-classifier were modest. This may be due to the fact that morphology-aware feature weighting adds further discriminative information to the already established temporal features of the conventional CNN–BiLSTM model.

### 3.4. Overall Results and Comparative Analysis

In order to evaluate the proposed ABMA framework for the arrhythmia classification task in depth, we conducted a series of experiments on the six different architectures that were introduced in this paper. All of the experiments for these architectures were conducted using a stratified 10-fold cross-validation protocol, and all of the architectures were run under identical experimental conditions. The feature extraction for each of the architectures was conducted using an identical pipeline of preprocessing steps, and all of the architectures were tested using an identical set of evaluation metrics as well as an identical set of optimization parameters. In particular, all of the architectures were tested using the exact same set of features that were normalized to have means of zero and standard deviations of one for each feature standardization, and the architectures were all tested using the exact same learning rate schedule as well as the exact same set of other training hyperparameters that controlled the optimization process. The results of these experiments are summarized in Table 5 for all of the architectures that were tested. The baseline CNN–BiLSTM architecture obtained a mean accuracy of 94.44% ± 2.73%, a weighted F1-score of 94.60% ± 2.54%, and a macro F1-score of 92.77% ± 2.27%. However, the lower value of the macro F1-score in comparison to the weighted F1-score is a sign of decreased robustness of the architecture for minority classes of arrhythmias (i.e., for AFIB and VT in particular). When we look at the results for all tested models, the complete ABMA framework is clearly the best model concerning the overall performance. The mean accuracy of the complete ABMA framework is 95.18 ± 1.18%, the weighted F1-score is 95.19 ± 1.18% and the macro F1-score is 94.66 ± 1.35%. We were able to improve the baseline model CNN–BiLSTM by 0.74 percentage points in terms of accuracy, by 0.59 percentage points in terms of weighted F1-score and by 1.89 percentage points in terms of macro F1-score. The lower standard deviations for all quality metrics also show that the ABMA framework is more robust than the baseline model. The standard deviations describe the spread of the single cross-validation runs and thus the ability of a model to generalize. In our case, the complete ABMA framework clearly shows a more consistent performance than the baseline model. Further insights into the performance of each individual component are obtained from the ablation analysis. Removal of the handcrafted feature branch led to a reduction in the network’s accuracy to 94.40 ± 1.42% and the corresponding macro F1-score to 93.80 ± 1.65%, which highlights the importance of the automatically derived physiological descriptors in conjunction with the deep ECG features learned through the network. Removal of the Morphology-Aware Attention module, on the other hand, yielded slightly lower results of 94.18 ± 1.33% accuracy and 93.57 ± 1.64% macro F1-score, since the Morphology-Aware Attention module enhances performance by learning morphology-aware importance scores for each time step that allow the network to focus on relevant portions of the ECG signal. Similar observations were made for the morphology scoring network, where scores calculated for morphology-aware importance enabled more discriminative features, although the results for accuracy (94.18 ± 1.45%) and corresponding macro F1-score (93.57 ± 1.81%) were comparable to those for the Morphology-Aware Attention module. The largest degradation among all the ablation variants was observed upon replacing the four-head attention mechanism with a single attention head. The resulting network’s accuracy dropped to 94.07 ± 1.28%, while its corresponding macro F1-score decreased to 93.36 ± 1.49%, which implies that the four individual attention heads of the four-head attention mechanism are able to learn distinct morphological representations that are not sufficiently captured by a single attention head. Our new framework, fully implemented as a complete ABMA model, even outperforms the individual class accuracies of the separately developed SA, AFIB and VT models, achieving F1-scores of 97.00 ± 0.76% (SA), 94.28 ± 1.54% (AFIB), and 92.70 ± 2.16% (VT) on the three classes. The effect of removing the handcrafted feature branch from the individual ablation models is smallest among all classes, while replacing the multi-head attention mechanism with a single attention head has the largest negative impact. The performance loss is especially pronounced for the VT class, where an F1-score of 90.64 ± 2.75% is achieved. Interestingly, while the individual ablation models already perform decently on all classes, their sensitivity to the removal of individual architectural components is much higher for classes with higher morphological and rhythm variability, i.e., AFIB and VT, than for the SA class. This confirms our design choice that the handcrafted feature branch, the morphology-aware attention mechanism, the morphology scoring network, and the multi-head attention mechanism in total and separately provide important information for a thorough classification of electrocardiogram signals. The results of the ablation study indicate that the highest accuracy, best balanced classification accuracy, and highest reliability of the ABMA framework are based on the combination of morphology-aware attention, the multiple representations that are learned by the model using the multiple- heads, and the additional physiological features. This synergy leads to better results than the sum of the single components.

Table 6 below shows the final set of hyperparameters that were used for training and evaluation of the proposed enhanced ABMA. These values were selected within the framework of a systematic search through candidate values, where all model settings were evaluated using the validation set within the cross-validation framework. The aim was to identify a hyperparameter configuration that provided a favorable balance between classification performance, training stability, model generalization, and computational efficiency. The ECG recordings were preprocessed in the following way: they were all of fixed length of 5000 samples (after padding as necessary), they were split into training and test sets using stratified 10-fold cross-validation, and they were trained using the Adam optimizer with an initial learning rate of 3 × 10^−4^. A warmup cosine decay schedule was used to train the model, as this allows the model to stabilize in a smooth way as it learns, rather than having it jump in a sudden way as the learning rate is decreased. Early stopping with a patience of 12 epochs was used to prevent overfitting. To capture ECG patterns at multiple temporal scales, the multi-scale CNN backbone consists of several convolutional layers with multi-scale kernel sizes (7 and 5) to extract local waveform characteristics across multiple temporal receptive fields. In addition, a BiLSTM layer with 64 units per direction is adopted to model the long-range rhythm dependencies. In the ABMA module, six attention heads are utilized to explore different morphology-aware representations from the ECG signals, and 96 hidden units are applied. Additionally, dropout is applied to all layers to improve the network’s generalization ability. To address class imbalance, all online data augmentation was performed on the training data. The amplitude of the training signals was mildly scaled, and additive Gaussian white noise as well as temporal shifts were introduced. The loss function used for optimizing the network was a class-balanced multi-class focal loss. This loss function highlights minority class samples in the loss function during training. No data augmentation or loss-weighting of categories was performed on the validation set or the test set. All performance measures are thus true measures of a model’s utility on unaltered (i.e., ‘clean’) data. The 10-fold cross-validation was performed in a stratified manner to ensure that the folds maintained the same ratio of majority to minority class samples. In turn, the ABMA framework was able to achieve strong and stable performance on the classification of ECG arrhythmias.

The model achieved better arrhythmia detection using convolution filter sizes of 7 and 5, which extracted both fine and coarse ECG features. The use of multi-scale feature extraction is essential for distinguishing arrhythmias. The early stopping mechanism was used to stop training when the validation performance stopped improving to prevent overfitting. The learning rate schedule was used to gradually decrease the learning rate, which helped in achieving better convergence and final accuracy. The hyperparameter tuning resulted in a robust classifier that achieved high accuracy on SA, AFIB and VT while being stable and not overfitting on this clinically complex dataset. To improve the diagnostic capabilities of the model, the addition of features such as ST segment elevation, PR interval and T wave amplitude is also considered. On the other hand, including many features may contain redundant or noisy information if these features are not well related to the target classes. This phenomenon is well documented in ECG signal analysis, where the challenge is to identify meaningful features for classification tasks [15]. The performance of models, especially in ECG classification tasks, depends heavily on feature selection. Studies have shown that the process of selecting ECG features is iterative, wherein several feature sets are evaluated to achieve the optimal classification performance. This challenge is inherently difficult because one is asked to estimate the performance of a feature set from ECG feature extraction without ever training and testing the classification model. As a result, ECG feature selection is an iterative process requiring the evaluation of multiple feature sets to achieve optimal classification performance. To classify arrhythmias, a new deep learning approach used 2D recurrence plot images of 2 s ECG segments. Stage 1 had 95.3% accuracy for noise or ventricular fibrillation detection, and Stage 2 reached 98.41% accuracy for atrial fibrillation, normal heart rhythms, early atrial fibrillation, and ventricular fibrillation after five-fold cross-validation. This method gives clinicians a reliable way to detect and differentiate arrhythmia types [32]. In [33], the authors’ study presents a 2D CNN approach for ECG arrhythmia classification. NOR, LBB, RBB, PVC and APC type ECG signals were transformed into the time frequency domain using short time Fourier transform and these spectrograms were fed into a 2D CNN to achieve an accuracy of 99% using MIT-BIH database. The best performance was achieved at the learning rate of 0.001 and batch size of 2500. The 2D CNN outperformed the 1D CNN with an accuracy of 90.93%, without the need for manual preprocessing of the ECG signal, making it suitable for ECG signal classification. In [34], the study is intended to contribute to the development of an ECG arrhythmia classification system using the Optimum Path Forest (OPF) classifier, a supervised graph-based technique, applied for the first time to the ECG signal classification. The OPF’s performance (training/testing time, accuracy, sensitivity and specificity) is compared with that of Support Vector Machines (SVM), Bayesian classifiers and Multilayer Perceptron (MLP) neural networks. From features of six common approaches in the ECG analysis and the MIT-BIH arrhythmia database, the OPF classifier showed robust, parameter-free performance, being faster and more accurate than MLP and SVM and as accurate as Bayesian classifiers. Thus, the OPF method can be considered as a promising, efficient tool for the ECG analysis with low computational cost. Authors [35] present the Deep Multi-Scale Convolutional Neural Network Ensemble (DMSCE) for robust arrhythmia classification. Using multiple scale-dependent DCNN expert classifiers with varying receptive fields, DMSCE simulates various pathological ECG characteristics. A convolutional gating network learns to compute fusion weights to aggregate local predictions from the experts, and a novel error function with a correlation penalty improves interaction and diversity among classifiers during training. On PTBXL-2020 (12-lead) and CinC-training2017 (single-lead) ECG datasets, the DMSCE achieved state-of-the-art F1-scores of 84.5% and 88.3%, respectively. Because of its strong generalization and scalability, it is suitable for remote and in-hospital cardiac monitoring. Authors [36] present a hybrid approach for ECG classification that combines deep learning features from AlexNet for both ECG and HRV. Using the Gabor transform, the ECG signals were transformed into 2D images and optimal features were extracted and classified to detect normal sinus rhythm, arrhythmia and congestive heart failure. The method has an accuracy of 98.75%, a specificity of 99.00%, a sensitivity of 98.18%, and a computation time of 0.15 s and thus has the potential for real-time clinical use. In [37], the paper proposes a spatiotemporal attention-based convolutional recurrent neural network (STA-CRNN) for improved arrhythmia detection from 12-lead ECG signals. STA-CRNN integrates CNN to extract spatial features, RNN for temporal information and attention modules to select relevant spatial and temporal information. The model obtained an average F1-score of 0.835 across 8 arrhythmia classes and normal rhythm and outperformed state-of-the-art methods on the same dataset. Visualization showed that the features learned by the STA-CRNN are consistent with clinical judgment and that it can support cardiologists in their arrhythmia diagnosis (refer to Table 4). In [16], the study discusses the difficulties of the automatic classification of ECG arrhythmias and the necessity of managing vast amounts of clinical data as well as irrelevant features. A two-step method was used: shallow feature extraction (time-domain analysis) and feature selection using a metaheuristic optimization algorithm. The experiment shows that choosing 1–3 features from RR interval assessment is sufficient to achieve perfect classification performance with 100% accuracy, sensitivity, specificity, and precision, and it is better than other techniques, suggesting that optimized feature selection is efficient for ECG analysis. Authors [38] have also used DL for classification of cardiac arrhythmia after reversing ECG images into time-series signals and digitizing ECG images. Using convolutional neural networks (CNN), long short-term memory (LSTM) networks, and self-supervised learning (SSL) models, ECG signals from digitized lead II heartbeats were classified. CNN had the highest accuracy of ~92%, which guarantees fast real-time inference. The proposed method also has the advantage of being able to integrate with ECG machines as a raw signal rather than an image, thus enabling real-time and accurate monitoring by cardiologists. Authors [39] present a deep learning-based approach for arrhythmia detection from ECG signals with minimal amount of preprocessing. A 1D CNN with a Gate Recurrent Unit (GRU) is employed for feature extraction and classification of five arrhythmia classes: Normal, PVCs, LBBB, RBBB, and paced beats. The method uses a multi-class Support Vector Machine (SVM) for extra classification, with an accuracy of 99.97%. The approach shows the possibility of using this approach for the efficient and accurate detection of arrhythmia in clinical applications with minimal data preparation. The proposed method demonstrates balanced performance across various arrhythmia classes by achieving 85% accuracy and 0.85 F1-score. The STFT-CNN [33] and AlexNet-Gabor [36] achieve higher accuracy rates of up to 99% but they require image transformations. The model preserves high interpretability and clinical relevance through its use of untransformed 12-lead ECG signals, and it does not require expensive transformations which makes it suitable for hospital system integration. The attention mechanism in ABMA provides interpretability whereas GRU-SVM hybrids [39] lack because their decision-making processes remain unclear (Refer to Table 7). To classify variable-length ECG signals, authors [40] employed a temporal attention mechanism with CNN and recurrent cells, achieving high accuracy of 81.2% while significantly improving the detection of paroxysmal arrhythmias. The proposed system also greatly reduces the complexity of computation and the number of model parameters. The authors present a multi-task channel attention network called MCA-Net using residual channel attention for the joint detection and localization of myocardial infarction from 12-lead ECGs. The network reaches a detection accuracy of >90% and localization accuracy of >80% on the dataset PTB-XL [41]. Yang designed CaMPNet, a multimodal transformer that employs cross-attention to integrate ECG signals, structured ECG features, and demographic data for the detection of cardiovascular comorbidities. CaMPNet achieved AUC values of 0.845 (on internal data) and 0.715 (on external data); however, its external generalization performance was poor [42]. Authors [43] present MS-LTCAF, a multi-scale lead-temporal co-attention framework that integrates lead-temporal co-attention and multi-scale feature extraction for multilead ECG arrhythmia classification. Experimental results on two datasets (PTB-XL and LUDB) achieved AUCs of 0.927 and 0.942, respectively, enhancing the lead–temporal representation. However, the results were not externally validated. The presented methodology, backed by thorough quantitative and visual analysis, also possesses strong potential for automation of clinical ECG analysis systems. In this work [44], we introduce a multibranch CNN, MB-CNN-ATT, for multilabel 12-lead ECG classification, where lead-wise attention fusion is further introduced to enhance inter-lead feature integration. Experimental results on two public datasets, PTB-XL and CPSC, show that MB-CNN-ATT outperforms state-of-the-art CNN- and ResNet-based methods with AUCs of 93.3% and 96.1%, respectively. In the paper, the authors introduced MB-CNN-ATT, a multibranch CNN for 12-lead ECG classification with lead-wise attention fusion. The proposed model outperforms state-of-the-art approaches in terms of AUCs (93.3% and 96.1%) and significantly enhances inter-lead feature learning [44]. In the paper [45], the authors propose a Multi-Scale Grid Transformer, called MSGformer, that integrates self-attention with multilead feature fusion and multi-scale grid attention for ECG classification. The approach yields an F1-score of 0.86 on CPSC 2018 and 99.28% accuracy on MIT-BIH arrhythmia database. The authors proposed [18] “Lead wise grouping multibranch network” that organizes 12-lead ECG network partitions into groups processed by parallel branches. State-of-the-art results are achieved with AUROC ~0.96 and F1 ~0.75–0.81. One of the main challenges is the strong lead dependency. The method significantly improves the efficiency of multi-label classification, and also some researchers have applied supervised machine learning techniques with hyperparameter tuning and SHAP interpretability in order to predict heart disease [19].

Differentiating between sinus rhythm disorders, atrial disorders, supraventricular disorders, and bundle branch and ventricular disorders using electrocardiography (ECG) can be challenging due to overlapping features and the complex nature of cardiac electrophysiology. Each category of arrhythmia presents distinct characteristics on an ECG, but subtle variations and similarities can complicate accurate diagnosis. Atrial disorders are those in which there is abnormal electrical activity of the atria [47]. AF is characterized by rapid, irregular atrial depolarizations resulting in an irregularly irregular ventricular response and the absence of distinct P waves on the ECG. Atrial flutter is another atrial disorder that shows a “saw-toothed” pattern of flutter waves at a rate of about 250–350 beats per minute, with a regular ventricular response depending on the degree of AV block. As AF and atrial flutter are easily confounded, distinguishing between the two is crucial because they have different clinical implications and management strategies. However, variable AV conduction or other coexisting conditions can make these patterns less clear [48,49]. Supraventricular disorders are arrhythmias originating above the ventricles, i.e., from the atria or the AV node. SVT is one of the most common types, which presents a fast heart rate with a narrow QRS complex on the ECG. The causes of SVT include mechanisms such as AV nodal reentrant tachycardia (AVNRT) or AV reentrant tachycardia (AVRT), each with its own slight ECG differences. The above conditions cannot be distinguished easily due to factors like rate dependent changes, pre-existing conduction abnormalities, or the effect of medications or electrolyte imbalance. For instance, atrial fibrillation with a rapid ventricular response can look like VT if there is a BBB, producing a wide QRS complex. Conversely, sinus tachycardia with aberrant conduction can look like SVT or VT. An accurate diagnosis is often made with clinical context, patient history, and other diagnostic tools like electrophysiological studies or advanced imaging. Although ECG is still a valuable method for diagnosing cardiac arrhythmias, the overlapping features of sinus rhythm disorders, atrial disorders, supraventricular disorders, bundle branch and ventricular disorders require a total and precise approach to interpretation. They necessitate an approach that is comprehensive and nuanced, even though ECG remains a fundamental tool in diagnosing cardiac arrhythmias. When necessary, consultation with electrophysiology specialists and continuing education and experience are essential for accurate diagnosis and management. Our proposed model demonstrated strong classification performance for both VT and AFIB, achieving high class-specific F1-scores across the cross-validation folds. The model’s performance and interpretability make it suitable for both acute care and routine monitoring settings if external validation confirms its robustness.

Misclassification between SA and AFIB exists, partly due to their overlapping characteristics and the difficulties inherent in accurately capturing and distinguishing the distinct morphological features of these two different arrhythmias. Both of these arrhythmias display irregular RR intervals; however, SA is distinguished by its organized P-waves and AFIB by the lack of P-waves. P-wave visibility has been found to be highly variable in quality due to factors such as noise, baseline wander, and variability in signal quality, resulting in some challenging instances for the model to classify correctly. This results in classification ambiguity in some cases and is reflected in the corresponding confusion matrix where there is considerable misclassification between SA and AFIB.

In order to get a deeper view of how individual components of the architecture contribute to the total performance of the ABMA framework, we perform a two-sided paired t-test on the mean of the individual folds of the complete ABMA and each of the comparison models (see Table 8). Importantly, we see that the complete ABMA outperforms the CNN–BiLSTM significantly in terms of all three measures (Accuracy, Weighted F1-score, and Macro F1-score), with *p*-values all less than 0.001. We also observe that, while accuracy, Weighted F1-score and Macro F1-score decrease consistently for all ablation models compared to the complete ABMA model, the decreases are not statistically significant at the 0.05 level. However, this observation in itself is very telling, because it implies that all components of the architecture of the ABMA framework, in isolation, contribute positively to its performance. Furthermore, the complete architecture of the framework, i.e., the sum of all individual components, delivers the best possible and most balanced results.

### 3.5. External Validation

**Figure 7:**
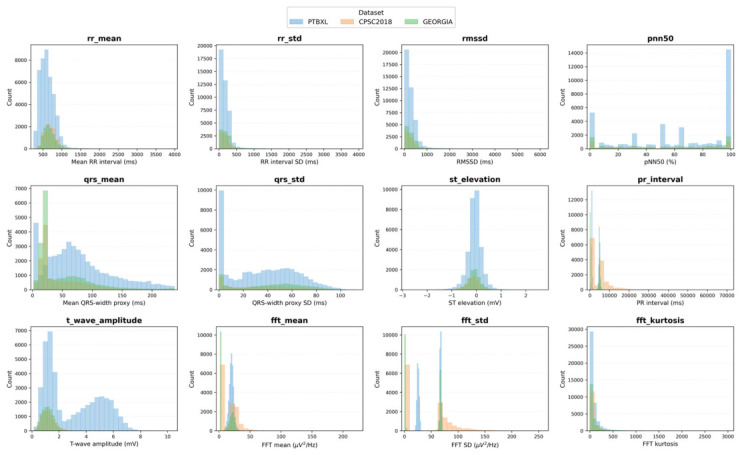
Distribution comparison of 12 extracted features between the internal training set and the 3 external datasets.

In order to test the generalizability of the ECG classification framework proposed in this paper, three publicly available datasets have been used for external validation. The results obtained for the classification task on the PTB-XL [33] dataset, the Georgia 12-lead ECG Challenge dataset [34] and the CPSC-2018 China Physiological Signal Challenge dataset [35] are summarized in Table 9. In all cases, the datasets used for external validation are very different from the internal training data used for the ECG classification framework proposed in this paper, in terms of patient demographics, in terms of the recording protocol, in terms of the environment in which the ECG signals were recorded and in terms of the available annotations for the observed signals and the corresponding clinical diagnoses. The external validation of a classifier is fundamental in order to test the ability of a model that has been trained on a given dataset to generalize to new, independent populations and to real-world applications in a clinical setting. In order to provide the reader with a qualitative understanding of the results of the external validation of the ECG classification framework proposed in this paper, the distributions of the handcrafted physiological features for the internal training data of the ECG classification framework proposed in this paper and for the three external datasets are plotted in Figure 7.

Figure 7 shows the distribution of the 12 handcrafted ECG features for the internal training set and the three external test sets. All features were standardized using the statistics of the internal training set, but there are still some distributional differences among the features. Features 4, 6, 7 and 10 have the largest differences among the features. Feature 10 has an even near-zero variance in all test sets, which means that it does not add any discriminative information in these test sets. Features 6 and 7, which are related to the ventricular depolarization features, have not only large-scale differences, but also large distribution differences. These large differences indicate the large morphological differences and also the large differences in the acquisition conditions of the ECG signals. These large distribution differences among the datasets in this study are the main reason for the large covariate shift and the resulting large decrease in the external classification accuracy.

The model achieved strong results in terms of internal validation performance (see Accuracy and class-specific F1-scores for SA, AFIB and VT above). However, when we deployed the model to the external test sets, performance dropped somewhat. Of the three classes, performance for atrial fibrillation (AFIB) was the most stable, scoring an F1 of 0.88 on the Georgia test set (which, as previously noted, only contains recordings of AFIB for this three-class classification task). The recalls for classification of sinus rhythm (SA) and for detection of ventricular tachycardia (VT) were 0.73 and 0.56, respectively. The Georgia test set only contains recordings of AFIB for this three-class classification task. Therefore, we are unable to report any results for SA or VT using this test set. In addition, using the CPSC-2018 test set, the model achieved an F1-score of 0.76 for classification of recordings of sinus rhythm (SA) while the F1-score for VT was a poor 0.03. This last result highlights a very serious problem for the minority class of recordings of VT, namely that the model fails to generalize to new recordings of this class very well.

There is clear evidence of distribution shift from internal training data to external data, as displayed in Figure 7 and demonstrated quantitatively by Table 8, Table 9 and Table 10 for all features with particular emphasis on Features 4, 6, 7 and 10. Feature 10 did not contain any information in all the external datasets with near-zero variance and thus was found to be of no discriminative power. This covariate shift or drift clearly led to the fall in classification accuracy of external data. However, for detection of AFIB, it performed consistently well across all the datasets in external data, which was largely due to the fact that the detection primarily relies on the rhythm of the ECG signal, and this feature is generally more consistent and has less variability across datasets than the morphology-dependent features.

While the model used in this paper performed extremely well in the internal validation of the used clinical datasets, the performance of the proposed framework is not yet sufficient to generalize to other datasets from different clinics, recorded in different ways and annotated by different people. In particular, the performance for the minority class ventricular tachycardia drops drastically on the CPSC-2018 external validation dataset. The big variance in performance for ECG classification between the different datasets in this paper is another illustration of the current challenge of cross-dataset classification and the many differences between the signals, the recording setup, the annotations and the patients of the various datasets.

While the current model can robustly differentiate VT from SVT with aberrant conduction, it may benefit from several clinically relevant features. These include the presence of AV dissociation and the QRS axis deviation, which can provide additional information to improve the ability to differentiate VT from normal beats and to achieve higher accuracy in discriminating between VT and SVT with aberrant conduction. The additional features, in particular the presence of AV dissociation, can be very powerful diagnostic indicators of VT. QRS axis can provide valuable information about the location of ventricular activation initiation and its propagation through the ventricle. However, reliable extraction of these features in clinical recordings often requires reliable P-wave detection and the analysis of multiple leads in different vector orientations, which can be affected by a lot of noise and signal variability. Future work will investigate the integration of these more advanced electrophysiological features into the proposed methodology, specifically into the morphology-aware attention framework, in order to improve clinical interpretability and the ability to distinguish between different cardiac arrhythmias.

### 3.6. Attention-Based Interpretability Analysis

**Figure 8:**
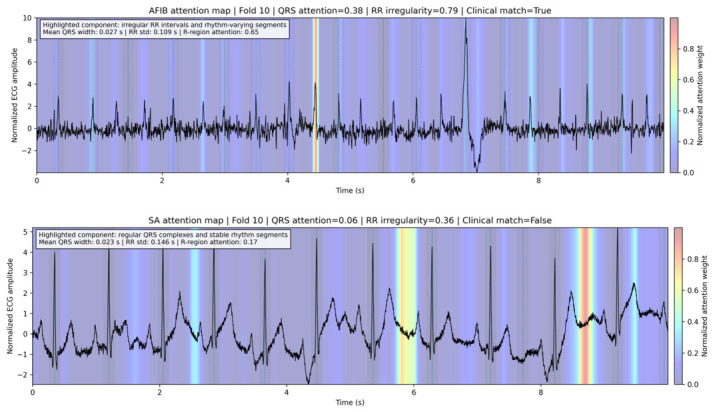
ECG attention maps for SA and AFIB, highlighting relevant clinical portions using the proposed ABMA module in ECG analysis.

Figure 8 shows representative examples of Lead II ECG signals and their respective attention maps that were generated by the ABMA module for the three heart rhythm classes SA, AFIB, and VT. The black line represents the normalized attention weights for a specific module within a channel, where high values are highlighted in warm colors. Green dashed lines mark the R-peak detection, and the corresponding gray shaded areas denote the QRS complex estimation. The highlighted parts in the respective regions of interest of the ECG examples qualitatively represent the relevant signal characteristics: The regular QRS complexes and the stable rhythm in SA, the highly variable and irregular RR intervals in AFIB, and the broad QRS complexes of ventricular origin in VT. Therefore, the model focuses on the respective most important ECG characteristics within the channels for classification purposes.

To demonstrate interpretability of the proposed ABMA, Figure 8 includes representative ECG attention maps obtained by the model for SA and AFIB classes. Color intensity of the attention maps represents normalized attention values. Higher intensity of warmer colors represents more important sections of the ECG signal that the model has used for classification of different heart rhythm classes. For the SA example, attention was spread relatively evenly across the regular QRS complexes and steady rhythm segments (even though the corresponding clinical judgment was relatively poor, because attention is not strongly focused on the QRS regions). In strong contrast, for the AFIB example, there was strong attention to the irregular RR intervals and the rhythm varying between segments, matching the hallmark features of atrial fibrillation. For example, in the VT case the attention is focused on the wide and repetitive ventricular QRS complexes that are typical for ventricular tachycardia. In this case the attention mechanism focuses on relevant parts of the ECG signal and avoids focusing on the same area in irrelevant parts of the signal. Note that we only provide attention maps as qualitative evidence for interpretability of our model and do not claim that they would give a full explanation for a model’s decision. We would need to validate our findings with the expert cardiologist annotations to confirm the relevance of the highlighted regions for clinical decision-making.

## 4. Conclusions

This paper proposes an Attention-Based Morphology-Aware (ABMA) framework that can classify sinus rhythm (SA), atrial fibrillation (AFIB), and ventricular tachycardia (VT) using ECG signals. The ABMA framework includes a hybrid deep learning model that combines multilead deep ECG representation with automatically extracted physiological descriptors. The framework, in particular, learns to extract rhythm dynamics and morphology-sensitive representations simultaneously, all without ECG delineation and/or without manual fiducial-point annotation. Local morphology extraction and multi-scale convolutional neural networks (CNNs) are jointly combined with temporal rhythm modeling using bidirectional long short-term memory (BiLSTM) networks and a novel Morphology-Aware Attention (ABMA) mechanism. The ABMA framework proposed in this paper has been evaluated and outperformed the baseline CNN–BiLSTM architecture. The contribution of each individual component of the proposed architecture has also been validated through a thorough ablation study. Specifically, the handcrafted features related to the physiological signal, the morphology-aware attention, the morphology scoring network and the multi-head attention mechanism have been proved to be all complementary and to increase the performance of the deep ECG features in the arrhythmia classification task. The combination of all the aforementioned components within the complete ABMA framework led to the best performance, in terms of accuracy and balance, among all the classes of interest. The main advantage of the proposed framework is represented by the fact that it exploits the morphology-aware temporal weights in combination with the handcrafted features that describe the physiological signal. This characteristic makes the deep ECG features, processed by the proposed architecture, more discriminative for the automatic classification of arrhythmias. We observed significant improvement in the performance of the framework on the challenging case of atrial fibrillation, a very irregular and variable arrhythmia. As the morphology-aware attention mechanism is able to focus on the most relevant time segments of the ECG signal, it is likely to capture additional ECG features that have not been captured by previous attention mechanisms, even though they might be relevant for classification. Therefore, the proposed morphology-aware learning approach is favorable for improving ECG analysis using deep learning methods. Although the results of this study are encouraging, there are limitations to this work. The study’s evaluation was carried out by using a three-class classification task on a curated dataset by means of a stratified internal cross-validation. Thus, the results obtained by using the proposed method in terms of performance must be considered as being confined to the employed dataset and the experimental protocol, which have been used for the evaluation. However, the obtained, robust and consistent results that were achieved by using the internal cross-validation on the used dataset are a good starting point for further validation in terms of robustness and generalizability by using independent external datasets, multicenter datasets as well as patient data. Future work aims to expand upon the current model by conducting a more in-depth external validation, as well as exploring the realms of domain adaptation, transfer learning, and multi-source training.

## Figures and Tables

**Table 1:** ECG feature extraction.

Descriptor Category	Feature Symbol	Mathematical Definition
Temporal Rhythm	$F_{1}=RR_{mean}$	$\frac{1}{N}\sum_{i=1}^{N}RR_{i}$
	$F_{2}=RR_{std}$	$\sqrt{\frac{1}{N}\sum_{i=1}^{N}(RR_{i}-RR_{mean}{)}^{2}}$
	$F_{3}=RR_{rms}$	$\sqrt{\frac{1}{N}\sum_{i=1}^{N}RR_{i}^{2}}$
	$F_{4}=RR_{irreg}$	(\displaystyle \frac{1}{\max(1,N−1)}\sum_{i=1}^{N−1}\mathbb{I})
QRS-Width Proxy	$F_{5}=Width_{mean}$	Mean of peak widths measured at half-maximum amplitude (FWHM)
	$F_{6}=Width_{std}$	Standard deviation of FWHM peak widths
Global Spectral	$F_{7}=FFT_{mean}$	Mean of FFT magnitude spectrum
	$F_{8}=FFT_{std}$	Standard deviation of FFT magnitude spectrum
	$F_{9}=FFT_{skew}$	Skewness of FFT magnitude spectrum
	$F_{10}=FFT_{kurt}$	Kurtosis of FFT magnitude spectrum

**Table 2:** Demographic and clinical characteristics of the study cohort.

Characteristic	SA	AFIB	VT	Overall
Number of ECG recordings, n (%)	2671 (71.4%)	804 (21.5%)	265 (7.1%)	3740 (100%)
Age (years), mean ± sd	33.05 ± 23.52	62.66 ± 16.85	71.97 ± 10.66	42.17 ± 26.01
Male, n (%)	1335 (50.0%)	454 (56.5%)	159 (60.0%)	1948 (52.1%)
Female, n (%)	1336 (50.0%)	350 (43.5%)	106 (40.0%)	1792 (47.9%)
Sampling frequency (hz)	500	500	500	500
Signal length (samples)	5000	5000	5000	5000

**Table 3:** Baseline model performance across folds, classes, and computational efficiency.

Fold	Accuracy (%)	Weighted F1 (%)	Macro F1 (%)	SA F1 (%)	AFIB F1 (%)	VT F1 (%)	Training Time (s)	Inference Time (s) per Fold
1	96.52	96.53	94.90	98.04	93.62	93.04	3878.42	1.75
2	91.44	91.76	89.34	94.12	81.63	92.28	2721.36	1.89
3	96.25	96.27	94.32	97.66	92.31	92.98	2916.58	1.82
4	95.19	95.24	93.17	96.88	90.74	91.89	4983.17	1.84
5	94.39	94.47	93.61	96.49	88.89	95.45	3506.92	1.78
6	93.85	93.94	91.95	95.89	87.80	92.16	2214.44	1.79
7	93.32	93.40	91.74	95.24	88.46	91.52	3460.55	1.87
8	93.58	93.66	91.21	95.65	87.34	90.65	3528.21	1.81
9	95.72	95.78	94.17	97.53	91.43	93.54	2597.83	1.90
10	94.12	94.26	93.28	95.43	90.38	93.82	4492.12	1.91
Mean ± SD	94.44 ± 2.73	94.60 ± 2.54	92.77 ± 2.27	96.69 ± 2.11	87.26 ± 7.22	91.93 ± 3.42	3429.96 ± 1059.49	1.83 ± 0.20

**Table 4:** Performance of the ABMA model across folds, including overall, class-wise, and computational metrics.

Fold	Accuracy (%)	Weighted F1 (%)	Macro F1 (%)	SA F1 (%)	AFIB F1 (%)	VT F1 (%)	Training Time (s)	Inference Time (s)
1	95.09	95.10	94.55	96.99	94.21	92.44	1188.97	2.41
2	94.18	94.15	93.40	97.06	92.22	90.91	864.81	2.45
3	96.36	96.37	96.12	97.63	94.97	95.76	1450.74	2.45
4	97.27	97.27	96.91	98.02	97.77	94.96	1438.68	2.49
5	96.00	96.01	95.52	97.81	94.92	93.83	705.84	2.39
6	94.00	94.08	93.41	96.13	93.48	90.63	721.21	2.43
7	95.45	95.48	94.89	97.38	94.74	92.56	817.51	2.50
8	95.09	95.09	94.90	96.05	94.08	94.56	1439.13	2.46
9	93.26	93.26	92.40	95.87	92.53	88.80	700.54	2.52
10	95.08	95.05	94.50	97.09	93.84	92.56	679.01	2.42
Mean ± sd	95.18 ± 1.18	95.19 ± 1.18	94.66 ± 1.35	97.00 ± 0.76	94.28 ± 1.54	92.70 ± 2.16	1000.65 ± 338.74	2.45 ± 0.04

**Table 5:** Overall performance comparison across models.

Model	Accuracy (%)	Weighted F1 (%)	Macro F1 (%)	SA F1 (%)	AFIB F1 (%)	VT F1 (%)	Training Time (s)
Full ABMA	95.18 ± 1.18	95.19 ± 1.18	94.66 ± 1.35	97.00 ± 0.76	94.28 ± 1.54	92.70 ± 2.16	1000.65 ± 338.74
No Handcrafted Features	94.40 ± 1.42	94.39 ± 1.42	93.80 ± 1.65	96.52 ± 0.78	93.25 ± 2.24	91.62 ± 2.36	844.34 ± 171.55
No Attention	94.18 ± 1.33	94.19 ± 1.31	93.57 ± 1.64	96.46 ± 0.87	92.92 ± 2.13	91.14 ± 2.86	841.86 ± 192.51
No Morphology Scorer	94.18 ± 1.45	94.16 ± 1.44	93.57 ± 1.81	96.37 ± 0.91	93.28 ± 2.68	90.99 ± 1.93	828.86 ± 149.66
Single-Head Attention	94.07 ± 1.28	94.06 ± 1.30	93.36 ± 1.49	96.31 ± 0.86	92.98 ± 2.02	90.64 ± 2.75	778.12 ± 107.82

**Table 6:** Final hyperparameter configuration of the proposed ABMA model.

Hyperparameter	Value	Justification
Initial learning rate	3 × 10^−4^	Provides stable optimization and smooth convergence during training
Optimizer	Adam	Efficient adaptive optimization for deep neural networks
Batch size	16	Balances computational efficiency and gradient stability
Dropout rate, Feed forward and classifier dropout	0.1, 0.2 and 0.4	Reduces overfitting and improves model generalization
Attention heads	4	Enables learning of complementary morphology-aware feature representations
CNN kernel sizes	7, 5	Captures ECG waveform characteristics at multiple temporal scales
Bilstm units	64 per direction	Models long-range temporal dependencies and rhythm irregularities
Morphology scoring hidden units	96	Provides sufficient capacity for learning morphology-sensitive importance weights
Early stopping patience	12 epochs	Prevents overfitting while allowing stable convergence
Learning rate schedule	Warmup Cosine Decay	Improves optimization stability and training convergence
Random seed	42	Ensures experimental reproducibility
Loss function	Focal Loss with Class Weights	Improves learning for minority and difficult classes

**Table 7:** Comparison report on the state of the art along with our approach.

Ref	Approach	Dataset	Performance (%)	Highlights	Issues
[37]	Spatiotemporal Attention CRNN (STA-CRNN)	12-lead ECG	83.5	Combines CNN, RNN, attention for improved detection	The growing complexity of architecture creates additional computational requirements that prevent real-time deployment unless optimization techniques are applied. The improved interpretability needs clinical validation to establish trust and real-world usage among practitioners.
[40]	ATI-CNN	ECGs (9 arrhythmia classes)	Accuracy: 81.2%	To detect paroxysmal arrhythmia detection	Moderate overall accuracy, no morphology-aware attention
[41]	CNN and Channel Attention	12-lead ECG	90% Accuracy (PTB & PTB-XL)	Channel attention exploits inter-lead relationships	The method was designed specifically for the task of myocardial infarction detection and not multi-class arrhythmia classification
[42]	CaMPNet (Multimodal Transformer + Cross-Attention Fusion)	MIMIC-IV ECG	Mean AUC 0.845, External: Mean AUC 0.715	Integrates raw ECG, structured ECG features, using cross-attention	External performance declines due to temporal distribution shift
[43]	MS-LTCAF (Multi-Scale Lead-Temporal Co-Attention Framework)	12-lead ECG	AUC 92.7; LUDB: AUC 94.2, Accuracy 92.0, F1-score 74.5	Lead-temporal co-attention with multi features	No external multi-center validation and architectural complexity and computational cost
[44]	MB-CNN-ATT	PTB-XL and CPSC	AUC: 93.3% (PTB-XL), 96.1% (CPSC)	Lead-wise attention fusion	no external multi-center validation
[45]	MSGformer	CPSC 2018, MIT-BIH	Accuracy: 99.28%, Sensitivity: 97.13%,	Transformer-based architecture with self-attention, multi-head	Lacks morphology-aware feature fusion
[18]	Lead wise grouping multibranch network	12-Lead ECG	AUROC ~0.96	multi-label classification performance	Dependence on lead grouping strategy
[46]	1DCNN	12-Lead ECG	accuracy: 98%	Real-time and clinical deployment	Sensitive to noise and baseline wander in ECG signals
Ours	Morphology-Aware Attention (ABMA) with CNN	12-lead ECG	95.9 (Accuracy), 0.9597 F1	Custom layer: Attention-Based Morphology	Requires external validation on multi-center datasets; slight sensitivity to class imbalance

**Table 8:** Results of paired two-sided *t*-tests comparing fold-wise performance metrics between the ABMA and Baseline models.

Comparison	Metric	Mean Difference (%)	t-Value	*p*-Value	Significance
ABMA vs. Baseline	Accuracy	+1.26	5.42	<0.001	Significant
	Weighted F1-score	+1.18	5.97	<0.001	Significant
	Macro F1-score	+1.16	6.80	<0.001	Significant
ABMA vs. No Handcrafted Features	Accuracy	+0.84	1.61	0.142	Not Significant
	Weighted F1-score	+0.85	1.65	0.133	Not Significant
	Macro F1-score	+0.93	1.57	0.151	Not Significant
ABMA vs. No Attention	Accuracy	+1.06	2.01	0.076	Not Significant
	Weighted F1-score	+1.05	2.03	0.073	Not Significant
	Macro F1-score	+1.15	1.89	0.091	Not Significant
ABMA vs. No Morphology Scorer	Accuracy	+1.05	2.17	0.059	Not Significant
	Weighted F1-score	+1.07	2.20	0.056	Not Significant
	Macro F1-score	+1.22	2.24	0.051	Borderline
ABMA vs. Single-Head Attention	Accuracy	+1.16	2.00	0.076	Not Significant
	Weighted F1-score	+1.17	2.05	0.071	Not Significant
	Macro F1-score	+1.31	2.03	0.073	Not Significant

**Table 9:** External validation classification report.

Dataset	SA Precision	SA Recall	SA F1-Score	AFIB Precision	AFIB Recall	AFIB F1-Score	VT Precision	VT Recall	VT F1-Score	Overall Accuracy
Training	1	0.96	0.98	0.9	0.99	0.94	0.96	0.87	0.91	0.96
Ptb-xl	0.91	0.73	0.81	0.83	0.95	0.88	0.7	0.56	0.62	0.84
Georgia	Zero Samples	Zero Samples	Zero Samples	1	0.95	0.98	Zero Samples	Zero Samples	Zero Samples	0.95
Cpsc-2018	0.97	0.62	0.76	0.53	0.87	0.66	0.01	0.5	0.03	0.7

**Table 10:** Name ECG features.

Feature ID	Feature Name	Description	Clinical Relevance
Feature 1	Rr mean	Mean of rr intervals	Overall heart rate estimation
Feature 2	Rr std	Standard deviation of rr intervals	Heart rate variability (hrv)
Feature 3	Rmssd	Root mean square of successive rr differences	Detects rhythm irregularity (afib)
Feature 4	Pnn50	Percentage of rr differences > 50 ms	Hrv indicator (afib, sa)
Feature 5	Qrs mean	Mean qrs duration	Differentiates wide vs. narrow complexes (vt vs. sa/afib)
Feature 6	Qrs std	Variability in qrs duration	Detects conduction abnormalities
Feature 7	St elevation	St segment deviation (elevation/depression)	Ventricular repolarization abnormalities
Feature 8	Pr interval	Time between atrial and ventricular activation	Av conduction (short/absent in afib)
Feature 9	T-wave amplitude	Magnitude of t-wave	Repolarization abnormalities
Feature 10	Fft mean	Mean of frequency spectrum	Global rhythm characteristics
Feature 11	Fft std	Standard deviation of spectrum	Signal complexity/variability
Feature 12	Fft skew/kurtosis	Higher-order spectral statistics	Detects irregular/non-gaussian rhythms

## Data Availability

PTB-XL, a large publicly available electrocardiography dataset at https://physionet.org/content/ptb-xl/1.0.3/ (accessed on 14 November 2024).
